# Corneal Backscatters as an Objective Index for Assessing Fuchs' Endothelial Corneal Dystrophy: A Pilot Study

**DOI:** 10.1155/2017/8747013

**Published:** 2017-07-02

**Authors:** Hsueh-Yen Chu, Ching-Hsi Hsiao, Phil Yeong-Fong Chen, David Hui-Kang Ma, Chee-Jen Chang, Hsin-Yuan Tan

**Affiliations:** ^1^Department of Ophthalmology, Chang Gung Memorial Hospital, Linkou, No. 5, Fuxing St., Guishan District, Taoyuan City 333, Taiwan; ^2^College of Medicine, Chang Gung University, No. 259, Wenhua 1st Rd., Guishan District, Taoyuan City 33302, Taiwan; ^3^Graduate Institute of Clinical Medical Science, Chang Gung University, No. 259, Wenhua 1st Rd., Guishan District, Taoyuan City 33302, Taiwan; ^4^Clinical Informatics and Medical Statistics Research Center, Chang Gung University, No. 259, Wenhua 1st Rd., Guishan District, Taoyuan City 33302, Taiwan

## Abstract

**Purpose:**

To provide an objective, quantitative approach for monitoring Fuchs' endothelial corneal dystrophy (FECD), with Scheimpflug imaging.

**Design:**

This is a retrospective case-control pilot study.

**Methods:**

The study group consisted of 53 eyes in 27 patients diagnosed with FECD, with normal subjects paired as control. Main outcome measures were corneal thickness, morphological patterns on densitograms, and indices of corneal density including the average area density (mean AD) and the average ratio of Descemet's membrane density versus area density (DM/AD) in Pentacam Scheimpflug images.

**Results:**

There were no significant differences in age and corneal thickness between FECD and normal groups. Morphologically, hanging-hammock patterns were noted on the densitograms of FECD patients, which were different from the high-back chair patterns in normal subjects. Quantitatively, mean AD and DM/AD were both elevated in FECD patients as compared with normal subjects (*P* = 0.01 and 0.025, resp.). In addition, FECD patients with corneal edema had significantly higher mean AD (*P* = 0.018) than those without corneal edema.

**Conclusions:**

This pilot study shows that Pentacam system provides an objective, quantitative way to approach FECD corneas. It can assist ophthalmologists in detecting the early change and in monitoring disease progression of FECD. Further studies are needed to consolidate the findings.

## 1. Introduction

Fuchs' endothelial corneal dystrophy (FECD), first described by Austrian ophthalmologist Ernst Fuchs [1], is a slowly progressing corneal disorder, characterizing as cornea guttae on the thickened Descemet's membrane (DM), generalized corneal edema, and decreased visual acuity. Exact pathogenesis of FECD remains unknown [2]. DM thickening was shown as a result of abnormal collagen band formation secondary to gene mutation histologically [3, 4]. Cornea guttae may cause debilitating glare when it forms into confluence, even when there is little to no stromal or epithelial edema and pachymetry is relatively normal [5]. And the Na-K ATPase pump site density in endothelial cells may be decreased progressively in FECD, resulting in stromal edema and eventually full thickness edematous opacity [6, 7]. Although the etiology of FECD is not fully understood, the genetic factors are suggested to be a major risk factor [7–9].

Usually, corneal endothelial abnormality in FECD may be observed before subjective symptoms develop [7]. Clinically, FECD can be diagnosed by dew-drops like central glittering brown corneal guttae in slit lamp biomicroscopy [7, 10] in conjunction with increased corneal thickness in pachymetry, decreased endothelial cell counts in specular microscopy, and hyporeflective areas representing guttae in endothelial background in confocal biomicroscopy [11]. A grading system to document progression of FECD was firstly described by Krachmer et al. [12]. The severity was graded based on the confluence and number of guttae and the presence of corneal edema. Most grading systems usually graded FECD into the stages with and without corneal edema and the stage with the presence of corneal scarring [13], while the determination of edema is mostly subjective. Although the diagnosis of FECD is rarely in doubt, stratifying the severity of disease is mostly subjective. For clinical staging, the subjective interobserver and intraobserver agreement is usually doubtful. Therefore, an objective clinical grading system may be valuable for monitoring the severity/progression of FECD, as well as for the timing for intervention.

In 2010, Shousha et al. [14] suggested custom-designed ultra-high-resolution anterior segment OCT (UHR-OCT) to be a new approach for FECD diagnosis because they found DM thickness increased in FECD patients compared with normal people. Repp et al. [15] have then provided an objective, potentially functional index based on pachymetric profile of the cornea. They demonstrated central to peripheral thickness ratio as an objective and repeatable metric for assessing the severity of FECD, which correlates to the function of endothelium, rather than morphology only. Although monitoring corneal thickness may be helpful for monitoring the progression of disease, it may not be an effective indicator for defining the severity of FECD at a single time point due to the variation of corneal thickness in normal population. And Wacker et al. [16] demonstrated abnormal posterior toricity in advanced FECD using Pentacam imaging system.

Pentacam (OCULUS Optikgeräte GmbH, Wetzlar, Germany) is a rotating Scheimpflug camera, providing 360 degrees, three-dimensional, high-resolution images of the anterior segment structures from the cornea to the lens in a short acquisition time [17]. It has been widely used clinically for preoperative evaluation of corneal refractive surgery [18], measurement of anterior chamber angle [19], evaluation of keratoconus [20], and other purposes. Point and area densitometry, a built-in software of Pentacam, displays results on the densitogram, enabling the measurements of structural backscatters in any desired points of the images taken. Lens densitometry, for example, provides an objective quantitative assessment by measuring the light backscatter of the crystalline lens with high repeatability [21, 22]. Recently, densitometry has been introduced for quantifying corneal opacities, as an optical index for corneal health, since the light backscatter is minimal in normal cornea. The intensity of corneal backscatters measured by corneal densitometry has been applied for evaluating various diseased conditions such as postoperative corneal haze after photorefractive keratectomy [18, 23], corneal opacity in bacterial keratitis, and corneal clouding in patients with mucopolysaccharidosis [24]. Any pathology leads to the change in corneal water content, collagen fiber diameter, spacing and orientation, and abnormal accumulation of macromolecules may all affect the light scattering effect in the cornea [25], affect light propagation into the eyes, and then possibly affect visual quality considerably [18, 26]. Therefore, the light backscatter of cornea detected by Pentacam corneal densitometry could be considered as an important and effective index in the analysis of various corneal diseases with repeatability and reproducibility proven in previous literatures, which indicates the optical health of cornea [27].

In this work, we aimed to evaluate the potential of Pentacam Scheimpflug densitometry as an objective, quantitative tool for approaching the morphological and optical alterations of cornea in FECD patients.

## 2. Materials and Methods

Institutional review board approval was obtained from the Chang Gung Medical Foundation, Taiwan. All data were collected from patients in the Chang Gung Memorial Hospital, Taiwan, during 2009–2012.

### 2.1. Study Population

Study group consisted of the 27 patients (53 eyes) diagnosed with FECD by slit lamp biomicroscopy combined with corneal specular microscopy. Although the severity of FECD was usually graded clinically on the basis of the area and confluence of guttae, and the presence of edema [15, 28], we divided eyes with FECD in our study into stages with and without the presence of corneal edema only, which were correlated to grades 0–4 and grade 5 in Krachmer's grading system, respectively. It was because the aim of our work was to analyze the light backscattering of cornea related to the presence of corneal guttae and corneal edema, rather than the progressive difference within mild and moderate stage. Control group comprised 27 normal subjects (53 eyes) receiving routine ocular examination that have normal slit lamp biomicroscopic findings.

### 2.2. Image Acquisition and Processing

**Figure 1:**
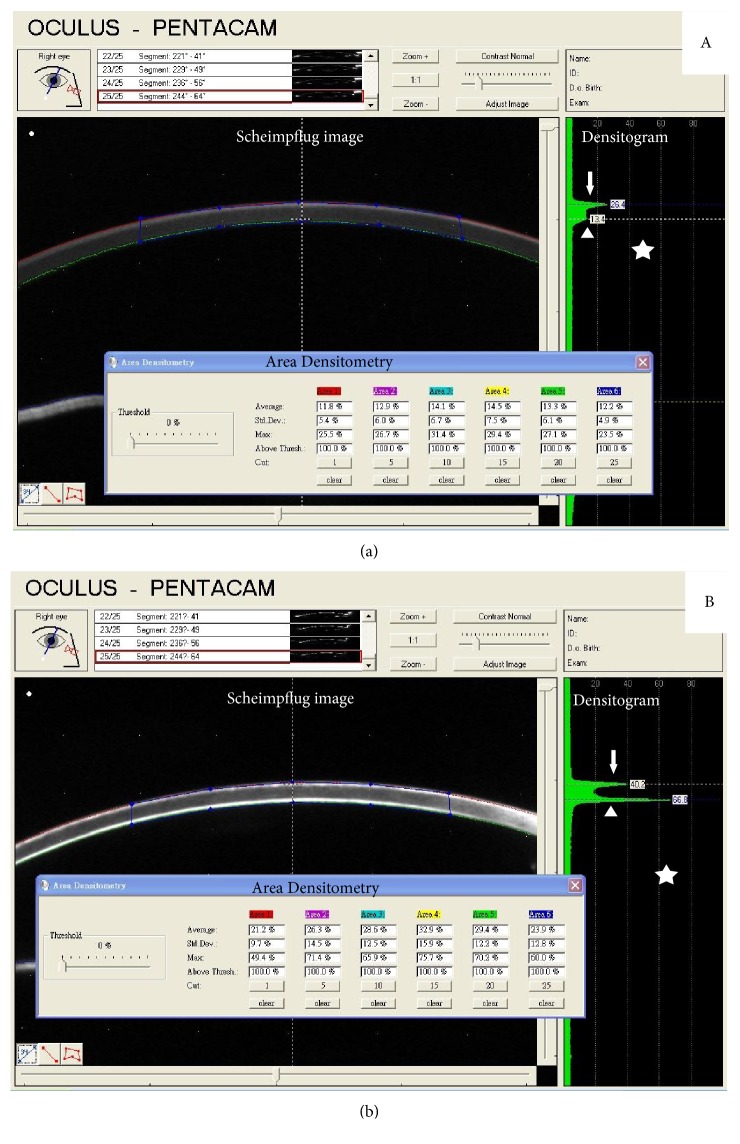
Densitometry of Pentacam Scheimpflug images. (a) One spiking hump with central flattening and a smoothing second hump appear as a high-back chair in normal subjects. (b) Two spiking humps look like a hanging hammock in FECD patients.

**Figure 2:**
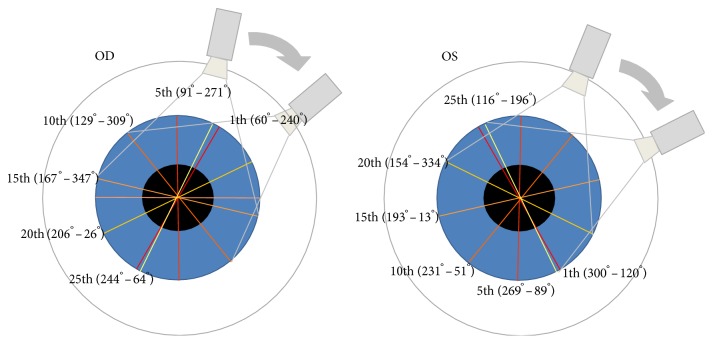
Basic settings of six selected segments in Pentacam.

All patients received the Pentacam examination under the same controlled ambient light conditions. Scheimpflug images were acquired automatically in 360 degrees fashion in the three-dimensional scan mode. Full-thickness corneal backscatter evaluation showed the measured maximum optical backscattering intensities on a densitogram on a relative scale from 0 to 100 (0 = no clouding, 100 = tissue completely opaque). A white marker line was positioned across the cornea and using the grid pattern superimposed on the image (Figure 1(a)). The backscatter intensity of the cornea was obtained at corneal apex (white dashed line in Scheimpflug image, Figure 1(a) left). The obtained readings of backscatter intensity were shown on the densitogram (Figure 1(a), right). Area densitometry program was initiated and shown as table in Figure 1(a). The central 4 mm full-thickness corneal cross-section in the Scheimpflug image was circled from apex (0 mm) to 2 mm centrifugally (Figure 1(a); blue-lined rectangle) to represent the corneal condition and early corneal change because the severity of FECD was greatest at the center initially. The first row of each column in the table of area densitometry revealed the average area density (intensity) (mean AD) in all six selected segments of rotating scanned Scheimpflug images, as shown in Figure 2, and were obtained with the threshold set at 0%. Central corneal thickness (CCT) values on the corneal topography were recorded. One left eye in both study and control groups was marked as missing data due to poor image acquisition.

### 2.3. Statistical Analysis

Statistical analysis was conducted using SPSS statistics 17.0 (SPSS Inc., Chicago, IL). To compare the difference between study and control groups, generalized estimating equation (GEE) was used. *P* < 0.05 was considered evidence of significance. To determine the relation of disease condition and CCT in the FECD patients, GEE was employed on all 53 eyes in the study group.

## 3. Results

### 3.1. Comparison of Corneal Backscatter between FECD and Normal Subjects

Of total 106 Scheimpflug image studies, 53 were obtained from 27 FECD patients (aged 41–89 years, mean 65 years). The other 53 were from 27 normal subjects (aged 42–85 years, mean 60.78 years). Mean CCT measured with Pentacam were 572.42 *μ*m in FECD patients and 546.62 *μ*m in normal subjects. GEE revealed no significant differences in age (Wald chi-square 0.002, *P* = 0.965) and in CCT (Wald chi-square 0.003, *P* = 0.954) between two groups. The insignificant difference in CCT can be explained by the individual anatomical variation superimposing on pathological changes in FECD.

**Figure 3:**
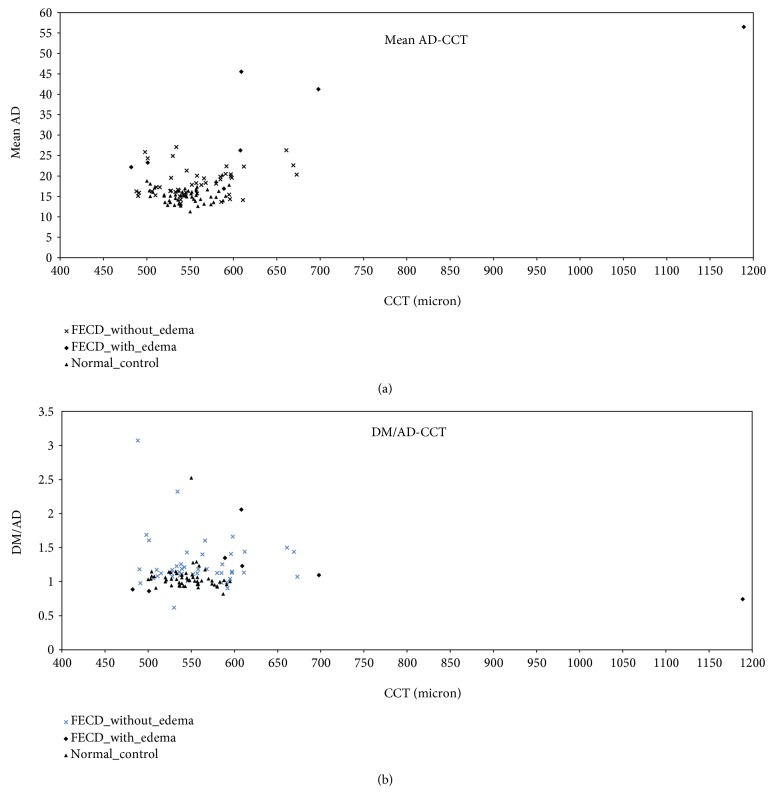
Scatterplots of mean area density (mean AD) against corneal thickness (CCT) (a) and average ratio of DM density versus area density (DM/AD) (b) in FECD without edema, FECD with edema, and normal eyes.

Representative corneal densitograms of normal subject (Figure 1(a)) and FECD (Figure 1(b)) were shown. Figure 1(a) (normal cornea) demonstrates only one spiking hump (white arrow) on the densitogram derived from epithelium backscattering with central flattening and a smoothing second hump (white arrowhead), appearing as a high-back chair. However, in Figure 1(b), a representative densitogram in FECD case, two spiking humps were noted with central depression, looking like a hanging hammock. The first hump on the densitogram in Figure 1(b) represented the backscattering of epithelium, while the second hump corresponded to that of the diseased DM in FECD cornea. Quantitative outcome measures were listed in Table 1. Mean AD is the average optical area density (intensity of backscattering effect) in the central 4 mm area of cornea. DM/AD is the proportion of DM backscattering effects on the central 4 mm corneal optical intensity. Mean AD and average ratio of DM density versus area density (DM/AD) in six selected segments were 20.37 and 1.25 in FECD group and 15.22 and 1.06 in normal control group, respectively. The data of mean AD as well as DM/AD against CCT in both FECD and normal groups was plotted in Figure 3. The values between groups differed significantly in mean AD (Wald chi-square 11.335, *P* = 0.01) and DM/AD (Wald chi-square 5.011, *P* = 0.025).

### 3.2. Comparison of Corneal Backscatter between FECD without Edema and Normal Subjects

Parameters as mean AD, DM/AD, and CCT were compared with GEE analysis for 99 eyes, including 53 normal eyes and 46 FECD with guttae alone eyes. It revealed no significant differences in CCT (Wald chi-square 0.270, *P* = 0.604), but significant difference in mean AD (Wald chi-square 14.611, *P* = 0.000) and DM/AD (Wald chi-square 4.552, *P* = 0.033) between normal eyes and FECD eyes without corneal edema.

### 3.3. Comparison of Corneal Backscatter between FECD Eyes with/without Corneal Edema

Parameters as mean AD, DM/AD, and CCT were compared for all 53 diseased eyes in the study group, which were further separated by clinical examination into guttae alone group (46 eyes in 24 patients) and edema group (7 eyes in 4 patients). GEE revealed no significant differences in CCT (Wald chi-square 0.261, *P* = 0.609) and DM/AD (Wald chi-square 0.144, *P* = 0.704), but significant difference in mean AD (Wald chi-square 5.559, *P* = 0.018) between guttae alone and edema groups.

## 4. Discussion

In this study, we used Pentacam densitogram to monitor the corneal backscattering effects in FECD patients. In qualitative analysis, the hanging-hammock pattern was observed on the densitograms in FECD patients, which was morphologically different from the high-back chair pattern in the normal subjects (Figure 1). Our findings were similar to the higher reflectivity, or “Camel sign,” on the densitograms of FECD patients reported by Renato Ambrosio et al., [29] who indicated that the second hump corresponded to corneal guttae at DM level. From the morphological pattern of densitogram, it showed that diseased DM contributes to marked increase in light backscattering of cornea.

### 4.1. The Difference of Corneal Backscatters between Normal and FECD Subjects

Quantitative analyses revealed significant elevation of mean AD and DM/AD in FECD patients as compared to those in normal subjects. Mean AD is the average optical area density in the central 4 mm area of cornea, which increases in FECD, regardless of the disease condition. As it represents the increase of corneal backscatter in FECD, it may therefore imply that the optical quality of transparent corneal tissue was affected in most of FECD patients, regardless of the presence of corneal edema.

DM/AD is the proportion of DM backscattering effects on the entire corneal optical intensity changes, which also elevates in FECD. This indicates that except the backscattering effect caused by corneal edema, pathological change in DM alone also contributes to the backscattering increase in FECD. Therefore, in the early stage of FECD, when pathological changes occur only in DM, backscattering also augments. This can contribute to impairment of optical quality of the cornea in the early stage of FECD when corneal edema is not observed clinically. We think that the increased backscattering of DM in FECD might be related to the pathological changes of DM. In the early-onset form of FECD, the anterior band layer of DM thickened, and an additional internal layer of wide-spacing collagen containing type VIII collagen within posterior nonband layer is demonstrated. An additional posterior striated layer rich in type VIII, which is not present in normal cornea, also contributes to the thickening of DM in early-onset form of FECD [4, 30]. In the classical late-onset form of FECD, the thickness of DM increases, with wide-spaced, irregular collagen deposited posterior to DM in form of posterior banded and fibrillar layers. Type VIII collagen was also found in the posterior fibrillar layer [30, 31]. We believe that both of the abnormalities in thickness and components might contribute to the change of optical properties of DM in FECD. Along with the disease progression, stromal edema and subepithelial fibrosis occur; the elevation of backscattering intensity in full thickness of cornea develops. We also compared normal eyes with FECD eye with guttae alone to exclude the effect of corneal edema in severe FECD eyes in causing the difference. The significant difference in mean AD and DM/AD again confirmed our observation that these two parameters can be used to help distinguish normal eyes from FECD eyes.

### 4.2. The Difference of Corneal Backscatters between FECD Patients with/without Corneal Edema

Within the FECD group, we noticed the elevation of the densitogram curve with less depression between double humps in corneal edema patients. Hence, we tested the relation between the presence of corneal edema and our proposed parameters: CCT, mean AD, and DM/AD. We found that DM/AD failed to show significant difference between guttae alone group and edema group. This may be because of the limited case number and the variety of edematous degree we collected in edematous group.

As for the relation between corneal edema and CCT, the result showed no surprisingly irrelevance. This not only explains the individual anatomical variation in CCT but also demonstrates the advantage of using Pentacam rather than corneal pachymetry in monitoring the disease progression of FECD.

The only parameter that differs significantly between guttae alone and edema groups is the increased mean AD in the latter. In swollen cornea, the uneven distribution of fluid, the change in collagen fibrils arrangement, and the change in refractive index result in the increase of scattering in the cornea, [32] which in turn augments mean AD. Hence, we can further infer that mean AD is a good indicator for disease progression.

In brief, in the early stage of FECD when the cornea is not edematous, diseased DM causes significant increase of corneal backscattering as compared to normal ones (different densitometric pattern, increased DM/AD, and increased mean AD). Along with the progression of FECD, corneal edema develops. The morphological pattern of corneal densitogram then changes, and the optical area backscattering augments (increased mean AD), which renders mean AD a better indicator than CCT considering the influence of optical quality in FECD corneas.

There are a few limitations in our work. Although Pentacam has already been equipped with advanced technology capable of producing high-resolution images, it is still not yet detailed enough in resolution compared to either spectral domain OCT or UHR-OCT. Nevertheless, Pentacam with its densitometry program quantifies the light backscattering effects, which approximates assessment of optical quality of cornea. Adding to that, Pentacam is more accessible in institutes or hospitals where corneal refractive surgeries are performed. This is a retrospective case-control pilot study. Visual acuity was not taken into analysis because its change involves multiple factors, including cataract condition. The Krachmer scale was not used to correspond our findings to it. Although the Krachmer scale is the most commonly used method to document the clinical severity in FECD, it is not always used by every clinician. While we were trying to test the validity of corneal backscatters measured by the Pentacam Scheimpflug imaging on assisting the FECD diagnosis, we could not have sufficient documentation with the Krachmer scale. Instead, we used the presence of corneal edema or not as a parameter to categorize FECD cases into “without edema, compatible with Krachmer's grade 0–4” and “with edema, compatible with Krachmer's grade 5.” Our work acts as a pilot study, trying to demonstrate the feasibility of the Pentacam Scheimpflug imaging as an objective and quantitative approach for FECD. Next, we will conduct a longitudinal study in order to assess the feasibility for corneal densitometry to monitor FECD patients. When we have a larger case number, we will try to identify specific cut-off values with receiver operating characteristic curve to determine sensitivity and specificity, to analyze the possible correlation between the 3D reconstructed Scheimpflug imaging of FECD corneas and Krachmer's grading, and to consolidate the validation of these parameters for detection or grading of severity of FECD.

In conclusion, we demonstrated that corneal backscatters measured by the Pentacam Scheimpflug imaging system can be provided as an objective, quantitative index for assessing optical health of the cornea in FECD. It not only potentially provides adjunctive diagnostic/monitoring information with stronger quantitative evidence but also enables assessment of the optical quality in FECD corneas with different disease conditions. As Pentacam is readily accessible and is a quick noninvasive examination with much information provided, the densitogram program can assist ophthalmologists in objectively and quantitatively detecting the morphological/optical changes in diseased corneas. And corneal backscatters can therefore be provided as an effective index for corneal optical health in various corneal pathologies. This is a pilot study. Further studies are needed to consolidate the validation of these parameters.

## Figures and Tables

**Table 1:** Quantitative parameters in FECD and normal subjects.

	FECD			Normal control		
	CCT (*μ*m)	Mean AD (%)	DM/AD	CCT (*μ*m)	Mean AD (%)	DM/AD
Numbers	23	23	23	23	23	23
Median	576	18.10	1.13	551	16.10	0.91
Minimum	511	13.65	0.74	505	13.13	0.79
Maximum	1189	56.48	2.32	667	20.07	1.10

FECD: Fuchs' endothelial corneal dystrophy; CCT: central corneal thickness; Mean AD: average area density; DM/AD: average ratio of Descemet's membrane density versus area density.
